# Loss‐of‐Function Mutations in SWI/SNF Complexes Associated With Active Tumour Immune Microenvironment Clinical Response to Immune Checkpoint Blockade

**DOI:** 10.1111/jcmm.71290

**Published:** 2026-08-25

**Authors:** Zheng Yang, Zixuan Tian, Renji Liang, Yi Lu, Tengfei Zhu, Ya‐nan Wang, Wei Zhang

**Affiliations:** ^1^ Department of Pathology The Seventh Affiliated Hospital of Sun Yat‐Sen University Shenzhen China; ^2^ Department of Medical Oncology Affiliated Hospital of Hebei University Baoding China; ^3^ Department of Cardiothoracic Surgery The First Affiliated Hospital of University of South China Hengyang China; ^4^ Department of Data Science Burning Rock Biotech Guangzhou China; ^5^ Department of Pathology Affiliated Hospital of Hebei University Baoding China; ^6^ Department of Pathology Beijing Hospital, National Center of Gerontology Beijing China

**Keywords:** biomarker, checkpoint blockade, loss‐of‐function mutations, SWI/SNF chromatin remodelling complexes, tumour microenvironment

## Abstract

Chromatin remodelling SWI/SNF complexes are recurrently altered in malignancies, and some members were found to be associated with immune checkpoint blockade (ICB) response. However, it is unclear whether SWI/SNF complex genes as a collective factor have clinical relevance. Through pan‐cancer analysis of 8507 tumours across 26 types from The Cancer Genome Atlas, we demonstrate that SWI/SNF loss‐of‐function (LOF) mutations correlate with elevated immunogenicity markers, such as higher tumour mutational burden (TMB), neoantigen load, and microsatellite instability. SWI/SNF‐LOF tumours exhibited enriched CD8^+^ T cell and M1‐like macrophage infiltration, upregulation of major histocompatibility complex class genes, and increased expression of immunostimulators, immunoinhibitors, and chemokines. Crucially, in ICB‐treated patients across discovery (*n* = 750) and validation (*n* = 562) cohorts encompassing nine cancer types, SWI/SNF‐LOF status consistently predicted superior clinical outcomes, including prolonged overall survival, progression‐free survival, and higher objective response rates compared to non‐mutated tumours. Multivariate analysis confirmed SWI/SNF‐LOF as an independent ICB response predictor independent of TMB, age, sex, or cancer type. These findings identify SWI/SNF LOF mutations as promising biomarkers of an inflamed tumour microenvironment and favourable responses to ICB across multiple solid tumours.

## 1 Introduction

In the past decade, immune checkpoint blockades (ICBs) targeting proteins such as cytotoxic T lymphocyte‐associated protein‐4 (CTLA‐4), programmed cell death protein 1 (PD‐1), and programmed death protein ligand‐1 (PD‐L1) have yielded promising results in the treatment of multiple types of solid tumours [1]. However, only 20%–40% of patients treated with ICB show a prolonged response, and the efficacy varies among different tumour types [1, 2, 3]. Due to the significant costs and potential for toxicity associated with ICB therapy, the identification of robust biomarkers capable of predicting individual clinical responses or resistance to such therapy has emerged as a crucial clinical challenge.

PD‐L1, Tumour Mutation Burden (TMB), and Microsatellite Instability‐High/Mismatch Repair Deficiency (MSI‐H/dMMR) have been approved as predictive biomarkers for immunotherapy by regulatory authorities. However, each of these biomarkers faces certain limitations and challenges. The use of PD‐L1 IHC as a biomarker is limited by variations in the quality and specificity of detection antibodies, differing standards and cut‐offs used for defining high or low PD‐L1 expression, the impact of tissue preparation on PD‐L1 expression, and substantial heterogeneity in PD‐L1 expression among various cancer types and tissues, including primary and metastatic tumours [4, 5]. Similarly, challenges associated with utilizing TMB as a predictive biomarker include difficulties in obtaining tissue samples, complex bioinformatic pipelines, and a lack of standardization in TMB assessment [6, 7, 8]. Additionally, the applicability and optimal cutoff values for TMB remain uncertain due to conflicting research findings [7, 8]. The use of MSI‐H/dMMR tumours as a predictive biomarker is restricted to select cancer types, and therefore has limited applicability [6, 9]. In summary, although PD‐L1, TMB, and MSI‐H/dMMR have shown potential as predictive biomarkers for immunotherapy, their clinical utility is hampered by technical hurdles, tumour response heterogeneity, and the absence of consensus regarding standardization and validation. Therefore, it is necessary to identify more biomarkers that are easy to measure, repeatable, and clinically relevant to predict ICB response. By identifying additional biomarkers that predict response to immunotherapy, researchers can potentially develop combination treatments that target multiple pathways and improve patient outcomes. This approach may also reduce the potential for resistance to single‐agent therapy. Therefore, the exploration of new biomarkers is essential for the advancement of precision medicine and the development of more effective immunotherapeutic strategies.

Studies have shown that a patient's genomic variation can affect many physiological processes, such as T cells, tumour cells and their interactions, and thus affect the efficacy of immunotherapy. For example, *POLE* and *POLD1* mutations have been proposed as biomarkers for the efficacy of immunotherapy in various types of cancer [10]. In addition, mutations in genes related to antigen presentation and interferon receptor signalling pathways, such as *B2M* and *JAK1/2*, have been linked to resistance to acquired immune checkpoint blockades (ICBs) [11]. The SWI/SNF family of chromatin remodelers has recently garnered attention as a potential correlate for clinical response to immunotherapy in various malignancies, including lung, colorectal, and kidney cancer [12, 13, 14, 15, 16, 17, 18, 19]. Previous research has suggested that SWI/SNF complexes likely function as tumour suppressors and regulate gene transcription and DNA damage repair by modulating chromatin accessibility [12, 20, 21, 22]. Recent studies have revealed that cBAF predominantly targets enhancer regions while PBAF and ncBAF target promoter regions [22, 23, 24]. Although the underlying mechanisms require further elucidation, preclinical evidence suggests that SWI/SNF inactivation is linked to increased TMB, CD8^+^ T cell count, DNA mismatch repair dysfunction (MMR), and heightened sensitivity to ICB in some cancer types [16, 25, 26]. Further research is needed to examine the relationship between the SWI/SNF inactivation, TMB, MSI status, homologous recombination deficiency (HRD), and immune microenvironment in different solid tumours.

Over the past 5 years, growing clinical evidence has linked response to immune checkpoint blockade (ICB) with mutations or loss‐of‐function (LOF) in several extensively studied SWI/SNF subunits, such as *PBRM1* in clear cell renal cell carcinoma [14], *ARID1A* in non‐small cell lung cancer (NSCLC) [17], colorectal [16], breast [18] and gastrointestinal cancers [15], and *SMARCA4* in lung cancer [13, 19]. However, previous studies investigating the SWI/SNF complex primarily focused on single genes or predefined sets of SWI/SNF genes, which may have led to biased conclusions due to the failure to account for all genetic variants [14, 17, 25, 26]. In addition, existing studies have focused on specific tumour types, and few studies have looked at pan‐cancer phenomena [13, 14, 15, 16, 17, 18, 19]. To address these gaps, we collected cohorts of patients treated with multiple ICB that had undergone whole‐exome sequencing (WES) in order to explore the association between SWI/SNF LOF mutations and several clinical phenotypes, including tumour mutational burden (TMB), tumour neoantigen burden (TNB), chromosomal instability, and response to ICB treatment. Compared to previous studies, our study investigates all 29 genes composing the SWI/SNF complex [12, 27], providing a broader perspective on gene function and its impact on ICB response. We anticipate that our findings will serve as a useful reference for molecular diagnostics and targeted drug development, stimulating further research into the complex molecular mechanisms of SWI/SNF LOF mutations and their relevance in predicting response to ICB therapy.

## 2 Methods

### 2.1 Patients and Data Acquisition

Data on the mutation profiles (sequenced by WES) and mRNA expression profiles of patients with 26 types of cancers in The Cancer Genome Atlas (TCGA) cohort, downloaded from the PanCancer Atlas consortium (https://portal.gdc.cancer.gov/), were used to investigate the distinct immune response landscapes of SWI/SNF LOF group and WT groups. As haematological malignancies, gliomas, and sarcomas are distinct in terms of their molecular features and biological behaviours, we excluded these malignancies from our analysis to ensure that our findings were specific to solid tumours.

To determine the predictive value of SWI/SNF loss‐of‐function (LOF) mutations in response to immune checkpoint blockades (ICBs) therapy, we employed a discovery and validation cohort. The discovery cohort included 750 patients from four publicly available cohorts who received ICB therapy. The cohort consisted of five cancer types, including bladder cancer (BLCA, *n* = 374), melanoma (*n* = 242), kidney renal clear cell carcinoma (KIRC, *n* = 72), non‐small cell lung cancer (NSCLC, *n* = 50), and head and neck squamous cell carcinoma (HNSCC, *n* = 12) [28, 29, 30, 31]. We also collected a validation cohort of 562 patients from seven cohorts who received ICB therapy. The validation cohort included four cancer types, namely NSCLC (*n* = 81), melanoma (*n* = 200), KIRC (*n* = 189), and gastrointestinal cancer (GI) (*n* = 92) [31, 32, 33, 34, 35, 36, 37]. Patients duplicated between discovery and validation sources were excluded from the validation cohort. To assess the predictive value of SWI/SNF LOF mutations for ICB response across multiple cancer types, we defined LOF mutations in SWI/SNF complexes as nonsense, frameshift, or splice‐site mutations.

### 2.2 Clinical Outcomes

The primary clinical outcomes were progression‐free survival (PFS), overall survival (OS) and objective response rate (ORR). Tumour response was assessed using the Response Evaluation Criteria in Solid Tumours 1.1. Durable clinical benefit (DCB) was defined as a response of complete response (CR), partial response (PR), or stable disease (SD) lasting ≥ 24 weeks, while no durable benefit (NDB) was defined as a response of progressive disease or SD lasting < 24 weeks. Objective response rate (ORR) was calculated as the proportion of patients with a CR or PR [34]. In the analysis of patients receiving immunotherapy, overall survival (OS) or progression‐free survival (PFS) were used to evaluate the efficacy of ICB therapy. For cohorts where a particular clinical endpoint had substantial missing data or insufficient follow‐up to allow robust survival analysis, that endpoint was excluded from the analysis.

### 2.3 Immunogenomic Indicators

Immunogenomic indicators for TCGA datasets were obtained from the pan‐cancer immune landscape project conducted by Thorsson et al. [38]. The indicators included TMB, MSI status, neoantigen prediction for SNVs and indels, intertumoral heterogeneity (ITH), homologous recombination deficiency (HRD), number of LOH segments and number of segments. The ITH was defined as the subclonal genome fraction determined from ABSOLUTE, while HRD score was calculated using three separate metrics of genomic scarring. The number of LOH segments is the number of segments with LOH events.

For samples sequenced with WES, TMB was calculated as the total number of nonsynonymous mutations divided by the exome size (38 Mb).

### 2.4 Immune Infiltration Analysis

CIBERSORT immune infiltration proportions, leukocyte fraction (measured using DNA methylation), stromal fraction (assessed using ABSOLUTE), and TIL regional fraction (assessed from TCGA H&E‐stained images) were obtained from the pan‐cancer immune landscape project conducted by Thorsson et al. [38]. Additionally, six immune expression signatures, including TGF‐β response and IFN‐γ response, were obtained from the same project.

For gene signature expression analysis, 149 immune‐related genes were collected and categorized into major histocompatibility complex class, receptor, immunoinhibitor, immunostimulator, and chemokine genes. These genes were sourced from TISIDB database (http://cis.hku.hk/TISIDB/data/immunomodulator.txt). Cytotoxic T lymphocyte genes, including *CD8A*, *CD8B*, granzyme A (*GZMA*), granzyme A (*GZMB*), and perforin 1 (*PRF1*), were also included in the analysis [39]. Expression levels were calculated as log2 (TPM + 0.001) to avoid taking the log of zero.

### 2.5 Statistical Analysis

Statistical analyses were performed using R software (version 4.0.3). Differences in categorical variables between groups were assessed using the chi‐square test, whereas differences in continuous variables were evaluated using the Mann–Whitney *U* test.

OS and PFS were estimated using the Kaplan–Meier method and compared using the log‐rank test. Hazard ratios (HRs) and corresponding 95% confidence intervals (CIs) were estimated using Cox proportional hazards regression models. An initial multivariable Cox model adjusting for age, sex, cancer type, and TMB level was fitted, and the proportional hazards assumption was assessed using Schoenfeld residual tests. Because the proportional hazards assumption was violated for cancer type, multivariable Cox proportional hazards models stratified by cancer type were subsequently used for the final survival analyses. TMB‐high was defined as the top 25% of TMB values within each cohort. For analyses involving multiple comparisons, *p* values were adjusted using the Benjamini–Hochberg false discovery rate (FDR) method where applicable. All reported *p* values were two‐tailed, and *p* < 0.05 was considered statistically significant.

## 3 Results

### 3.1 Patient Characteristics and Frequency of LOF Mutations in SWI/SNF Complex Genes

**FIGURE 1:**
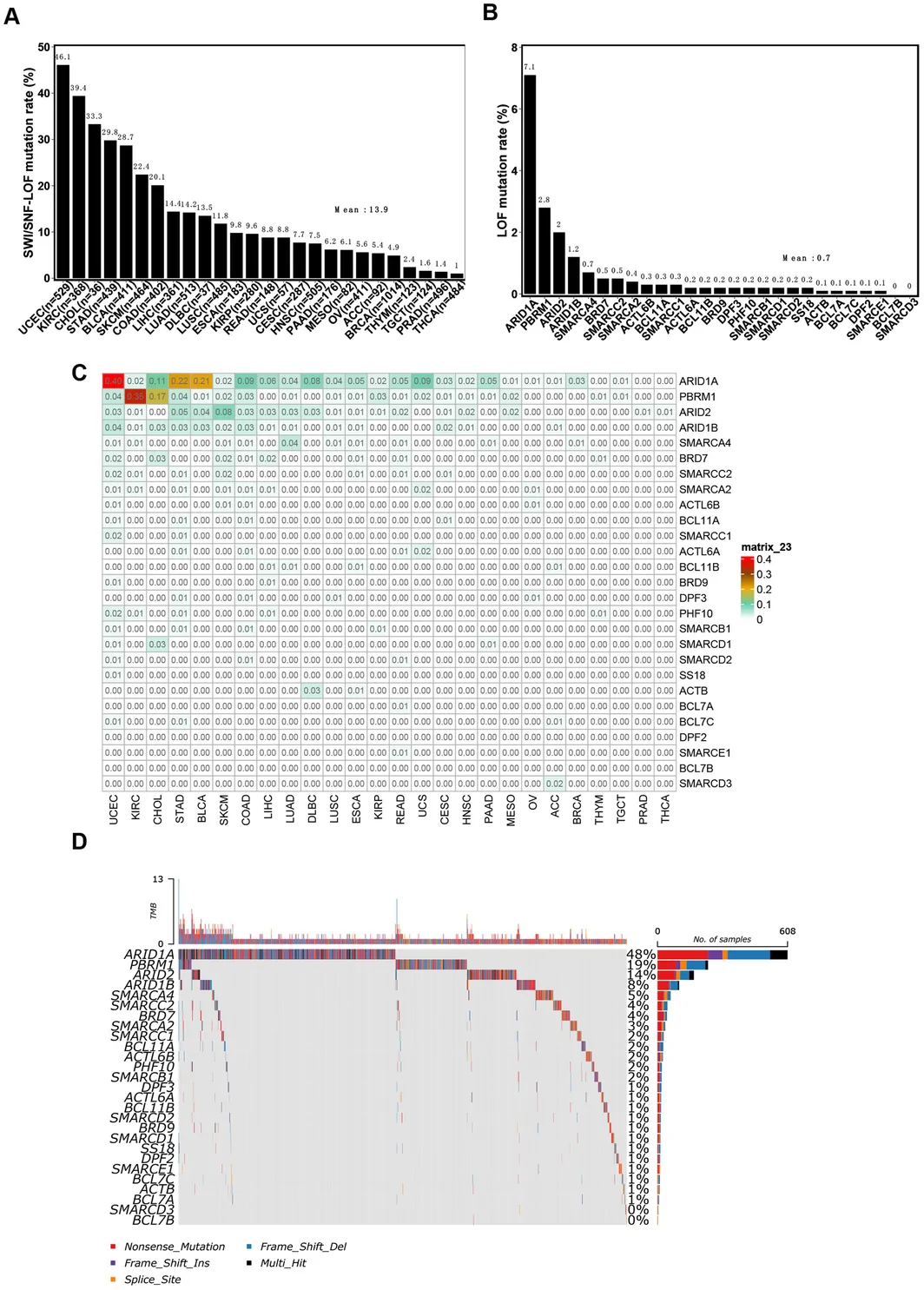
Loss‐of‐function (LOF) mutations in SWI/SNF complexes (SWI/SNF‐LOF) display tumour type‐specific distribution among different subunit‐encoding genes. (A) Frequency of SWI/SNF‐LOF among the 29 examined TCGA tumour cohorts. (B) Pan‐cancer LOF mutation frequency per gene. (C) Makeup of mutated subunits per tumour type. (D) A heatmap showing SWI/SNF‐LOF mutations in patients with ≥ 1 LOF mutations from the TCGA datasets. ACC, adrenocortical carcinoma; BLCA, bladder urothelial carcinoma; BRCA, breast invasive carcinoma; CESC, cervical squamous cell carcinoma and endocervical; CHOL, cholangiocarcinoma (bile duct); COAD, colon adenocarcinoma; DLBC, lymphoid neoplasm—diffuse large B‐cell lymphoma; ESCA, oesophageal carcinoma; HNSC, head and neck squamous cell carcinoma; KICH, kidney chromophobe; KIRC, kidney renal clear cell carcinoma; KIRP, kidney renal papillary cell carcinoma; LIHC, liver hepatocellular carcinoma; LUAD, lung adenocarcinoma; LUSC, lung squamous cell carcinoma; MESO, mesothelioma; OV, ovarian serous cystadenocarcinoma; PAAD, pancreatic adenocarcinoma; PCPG, pheochromocytoma and paraganglioma; PRAD, prostate adenocarcinoma; READ, rectum adenocarcinoma; SKCM, skin cutaneous melanoma; STAD, stomach adenocarcinoma; TGCT, testicular germ cell tumours; THCA, thyroid carcinoma; THYM, thymoma; UCEC, uterine corpus endometrial carcinoma; UCS, uterine carcinosarcoma; UVM, uveal melanoma.

This study included a total of 8507 patients from 26 different types of solid tumours in the TCGA cohorts. Loss‐of‐function (LOF) mutations in SWI/SNF complexes (SWI/SNF‐LOF) were identified as nonsense, frameshift, or splice‐site mutations. A total of 13.9% of the patients carried LOF mutations in at least one SWI/SNF complex gene (Figure 1A). However, no SWI/SNF‐LOF was observed in the kidney chromophobe, pheochromocytoma and paraganglioma (adrenal gland), and uveal melanoma cohorts, which were thus excluded from subsequent analysis.

Among the TCGA cohorts studied, seven tumour types showed SWI/SNF‐LOF mutation rates of greater than 20%. These included uterine corpus endometrial carcinoma (UCEC, 46.1%), kidney renal clear cell carcinoma (KIRC, 39.4%), cholangiocarcinoma (CHOL, 33.3%), stomach adenocarcinoma (STAD, 29.8%), bladder urothelial carcinoma (BLCA, 28.7%), skin cutaneous melanoma (SKCM, 22.4%), and colon adenocarcinoma (COAD, 20.1%) (Figure 1A). Across different cancer types, LOF mutations in *ARID1A* (7.1%), *PRRM1* (2.8%), *ARID2* (2.0%), and *ARID2* (1.2%) were the most common, while *BCL7B* and *SMARCD3* were the rarest (Figure 1B). There was also a tendency for cancer type‐specific occurrence of LOF mutations in individual subunit‐encoding genes (Figure 1C). For instance, *ARID1A* was the predominant mutant in bladder urothelial carcinoma and pancreatic adenocarcinoma, whereas in KIRC and CHOL the most mutated was *PBRM1*. The LOF mutations typically did not affect multiple SWI/SNF subunits, and nonsense mutations were the most prevalent type (Figure 1D). In terms of mutation type, nonsense mutations were the most prevalent type, and C>T transitions constituted a notable majority of the single‐nucleotide variations (Figure S1). There was no apparent clustering of mutated sites among the subunit‐encoding genes, which is consistent with the tumour‐suppressor role of SWI/SNF complexes (Figures [Link], [Link], [Link]). Moreover, patients in the SWI/SNF‐LOF group had higher age and tumour purity compared to those in the WT (non SWI/SNF‐LOF) group (Table 1).

**TABLE 1:** Demographic and clinical characteristics of patients from TCGA cohorts.

	Overall (*n* = 8507)	SWI/SNFLOF (*n* = 1256)	SWI/SNFWT (*n* = 7251)	*p*
Age (year)				
Median [IQR]	62.00 [52.00, 70.00]	64.00 [55.00, 72.00]	61.00 [52.00, 70.00]	< 0.001
Sex				
Female	4503 (52.9%)	655 (52.1%)	3848 (53.1%)	0.568
Male	4004 (47.1%)	601 (47.9)	3403 (46.9%)	
p_stage				
NA	2255 (26.5%)	340 (27.1%)	1915 (26.4%)	0.038
I/II	3922 (46.1%)	551 (43.9%)	3371 (46.5%)	
III/IIV	2330 (27.4%)	365 (29.1%)	1965 (27.1%)	
Purity				
Median [IQR]	0.62 [0.45, 0.78]	0.63 [0.47, 0.78]	0.62 [0.44, 0.78]	0.040

Abbreviations: IQR, interquartile range; SWI/SNF‐LOF, bearing 1 or more loss‐of‐function mutations in SWI/SNF subunits; WT, wild‐type.

### 3.2 Intrinsic Immune Landscapes in SWI/SNF LOF and WT Groups

In this study, we investigated the association between SWI/SNF LOF mutations and major factors in the innate immune response mechanism, such as high tumour immunogenicity and activation of antigen‐processing machinery (APM). Tumour types with a total sample size of less than 50 or LOF mutation frequency of less than 10% were integrated into “Others” tumour types for further analysis.

**FIGURE 2:**
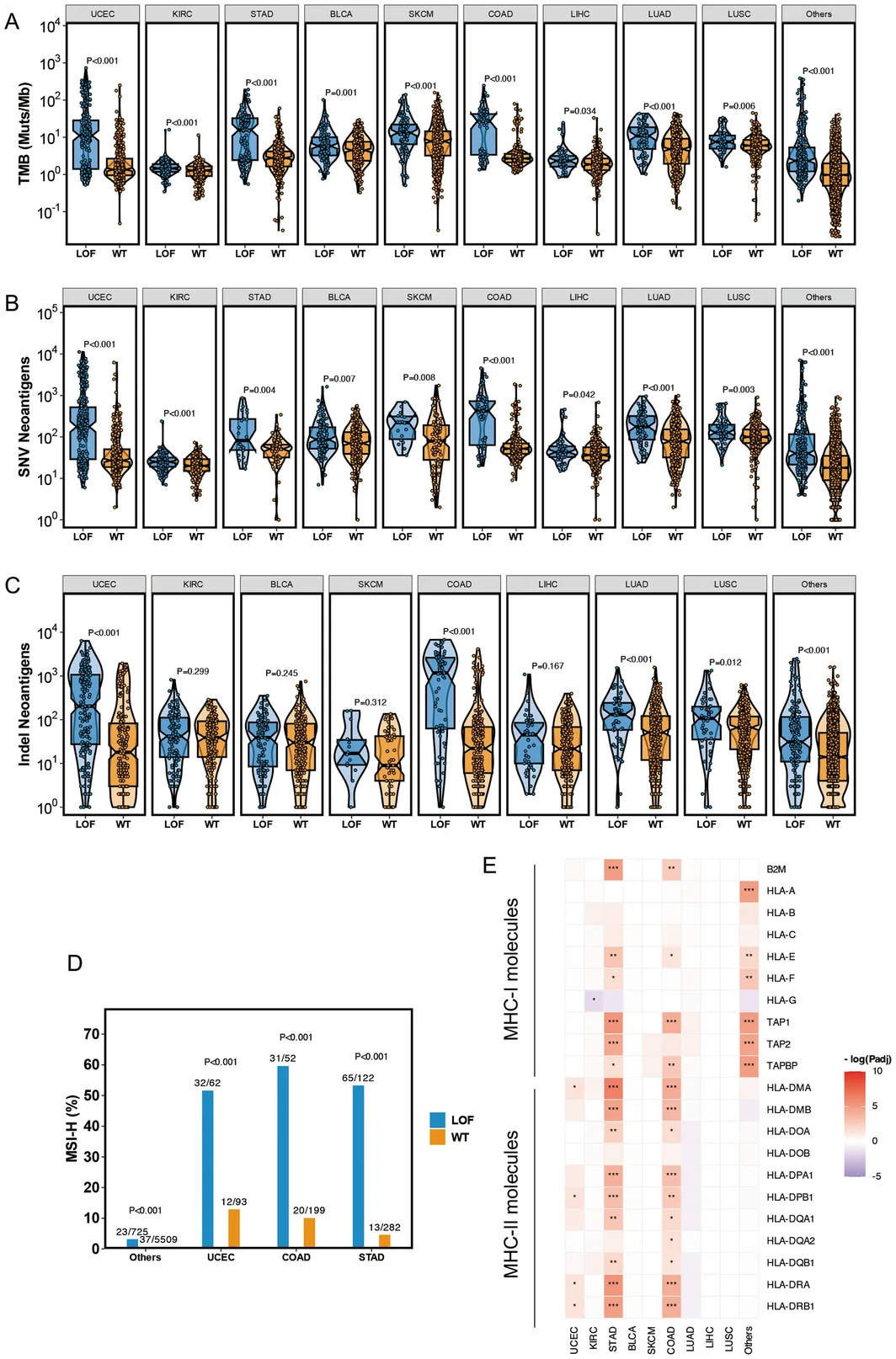
Relationship between SWI/SNF‐LOF mutations and immunogenicity markers in different cancer types based on TCGA cohort data. SWI/SNF‐LOF and wild‐type (WT) groups were compared in the following markers: (A) tumour mutational burden (TMB), (B) SNV neoantigen burden, (C) indel neoantigen burden, (D) microsatellite instability (MSI) status, and (E) major histocompatibility complex class gene expression.

We explored the relationship between SWI/SNF LOF mutations and several potential factors that determine tumour immunogenicity. The SWI/SNF LOF group consistently showed significantly higher TMB and SNV neoantigen load compared to the WT group (all *p* < 0.05) (Figure 2A,B). Although there was no significant difference in some malignant tumours, including KIRC, BLCA, SKCM, and liver hepatocellular carcinoma (LIHC), the SWI/SNF LOF group had a higher indel neoantigen load in general (Figure 2C). The MSI analysis involved a separate analysis of tumours with MSI characteristics, while the remaining tumours without MSI characteristics were combined into “Others” cohorts for analysis. We observed that the SWI/SNF LOF group exhibited a higher MSI ratio than the WT group in COAD, STAD, UCEC, and Others (all *p* < 0.05) (Figure 2D). These findings suggest that the association between SWI/SNF LOF mutations and increased tumour immunogenicity is not limited to specific types of tumours, but rather holds true across a range of malignancies. Moreover, the HRD score and CNV burden in the SWI/SNF LOF group were lower than the WT group in UCEC, STAD, and COAD, while the opposite was true for LUAD (Figure S5A,B). The number of LOH segments was also lower in the SWI/SNF LOF group than in the WT group in UCEC, KIRC, STAD, SKCM, and COAD, and the opposite was true for LUAD (Figure S5C). Previous research found that chromosomal instability is associated with immune avoidance markers [40]. This implies that WT group association between SWI/SNF LOF mutations and genomic instability may vary across tumour types.

Our analysis also involved comparing the expression levels of MHC‐related antigen‐presenting molecules between the SWI/SNF LOF and WT groups among various cancer types. The SWI/SNF LOF group expressed higher levels of MHC I‐related antigen‐presenting molecules in STAD, SKCM, COAD, and Others than the WT group (Figure 2E). Similarly, the SWI/SNF LOF group exhibited higher levels of MHC II‐related antigen‐presenting molecules in UCEC, STAD, and COAD when compared to the WT group (Figure 2E). These results suggest that SWI/SNF LOF mutations may contribute to enhanced tumour immunogenicity by upregulating the expression of MHC‐related antigen‐presenting molecules in most cancer types. Conversely, the lower expression levels of MHC‐related molecules in the WT group may contribute to intrinsic immune escape in several cancer types, suggesting that these tumours may be less responsive to immune‐based therapies.

### 3.3 Extrinsic Immune Landscapes in SWI/SNF LOF and WT Groups

**FIGURE 3:**
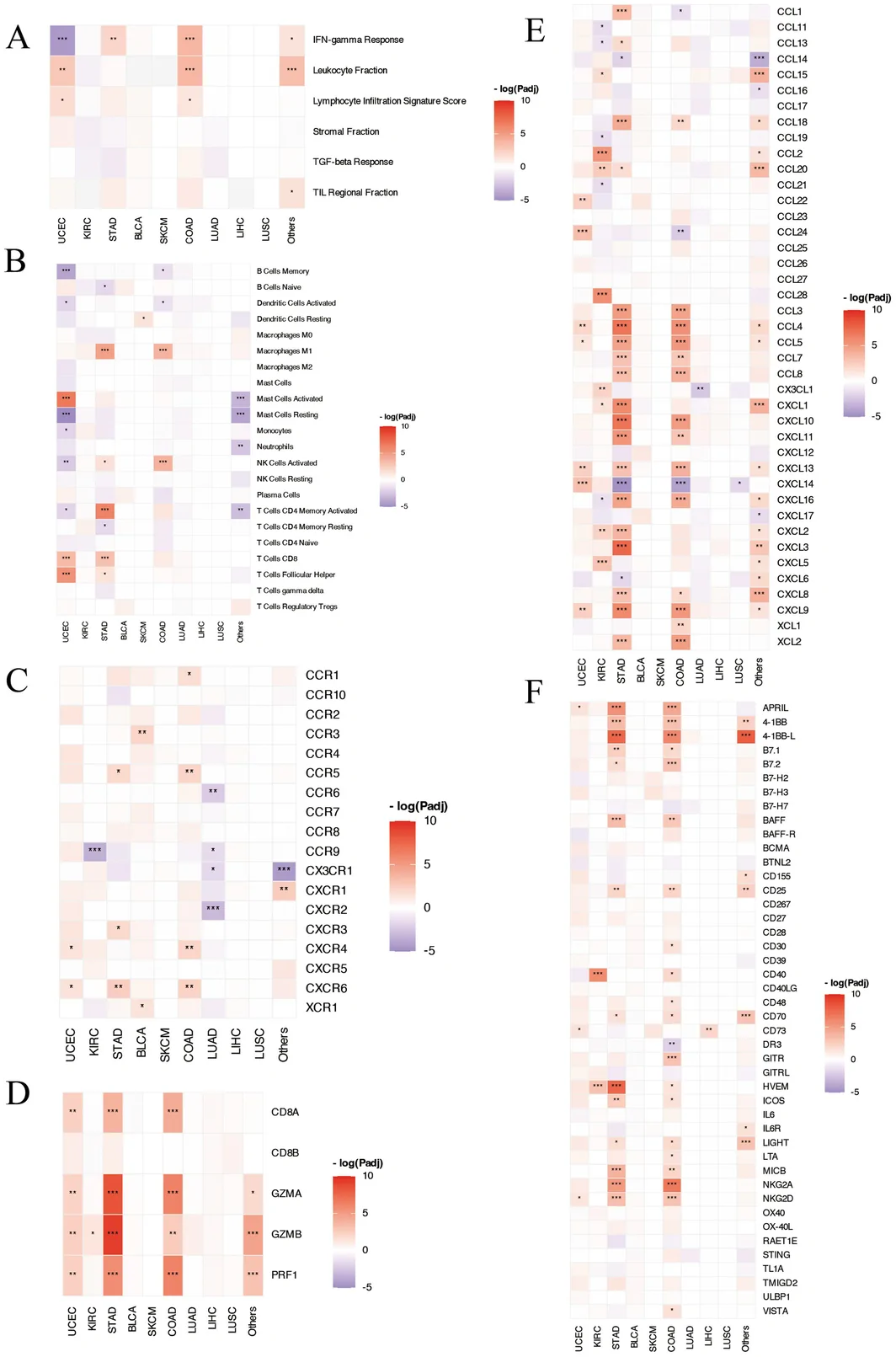
Potential intrinsic immune response and escape landscape in SWI/SNF‐LOF and WT groups in TCGA cohorts of different cancer types. (A) A comparison of leukocyte fraction, TIL regional fraction, lymphocyte infiltration signature score, stromal fraction, TGF‐β response, and IFN‐gamma response between SWI/SNF‐LOF and WT groups. (B) A comparison of the proportions of 22 types of immune cells estimated by the CIBERSORT method based on RNA‐sequencing data between SWI/SNF‐LOF and WT groups. (C, E, F) A comparison of the expression patterns of receptors, chemokines, and immunostimulators between SWI/SNF‐LOF and WT groups. (D) A comparison of the expression of cytolytic activity genes between SWI/SNF‐LOF and WT groups. The heatmaps illustrate differential levels of immune cell and immune‐related signature between the two groups, with red indicating higher values in SWI/SNF‐LOF and blue indicating the opposite.

To further investigate the relationship between the immune system and SWI/SNF LOF across multiple cancer types, we compared immune infiltration signatures and immune‐related genes between SWI/SNF LOF and WT tumours within each cancer type. After FDR correction, the SWI/SNF LOF group exhibited significantly higher leukocyte fraction in UCEC, COAD, higher lymphocyte infiltration signature scores in UCEC and COAD, and increased TIL regional fractions in Others compared with WT tumours (Figure 3A). IFN‐γ response signatures were elevated in STAD, COAD, and Others while reduced in UCEC, highlighting substantial tumour‐type‐specific heterogeneity in the immune context associated with SWI/SNF LOF mutations.

We next examined the 22 immune cell populations included in the original immune infiltration analysis. Following FDR correction within each cancer type, M1 macrophage infiltration was significantly enriched in STAD and COAD, whereas CD8^+^ T‐cell infiltration was increased in UCEC and STAD (Figure 3B). In addition, elevated expression of cytotoxic T lymphocyte‐associated genes was observed in SWI/SNF LOF tumours from UCEC, STAD, COAD, and Others (Figure 3D). These findings indicate that SWI/SNF LOF mutations are associated with an immune‐activated phenotype in selected tumour types.

Consistent with the immune infiltration patterns described above, SWI/SNF LOF tumours exhibited differential expression of chemokines and chemokine receptors after FDR correction (Figure 3C,E). Chemokines were more frequently upregulated in UCEC, KIRC, STAD, COAD, and Others. Higher expression of chemokine receptors was mainly observed in UCEC, BLCA, and COAD, whereas several receptors were downregulated in UCEC and Others. Together with the increased immune infiltration observed in selected tumour types, these results suggest that SWI/SNF LOF mutations are associated with altered immune signalling within the tumour microenvironment. We also examined the expression of immunostimulatory (Figure 3F) and immunoinhibitory (such as *PD‐L1*, *PD1*, *CTLA4* and *LAG3*) (Figure S6) molecules and found that these molecules were more highly expressed in the SWI/SNF LOF group than in the WT group. Immunostimulatory molecules were generally higher in SWI/SNF LOF tumours in UCEC, KIRC, COAD, STAD and Others, with only limited decreases observed. Immunoinhibitory molecules were also more frequently elevated in SWI/SNF LOF tumours, especially in UCEC, KIRC, COAD, STAD and Others. Overall, our comprehensive analysis of immune‐related factors in multiple cancer types demonstrates the relationship between SWI/SNF LOF mutations and immune cell infiltration and function but the specific effects may vary depending on the tumour type.

### 3.4 SWI/SNF‐LOF Was Related to Better Clinical Outcomes for ICB Treatment

**FIGURE 4:**
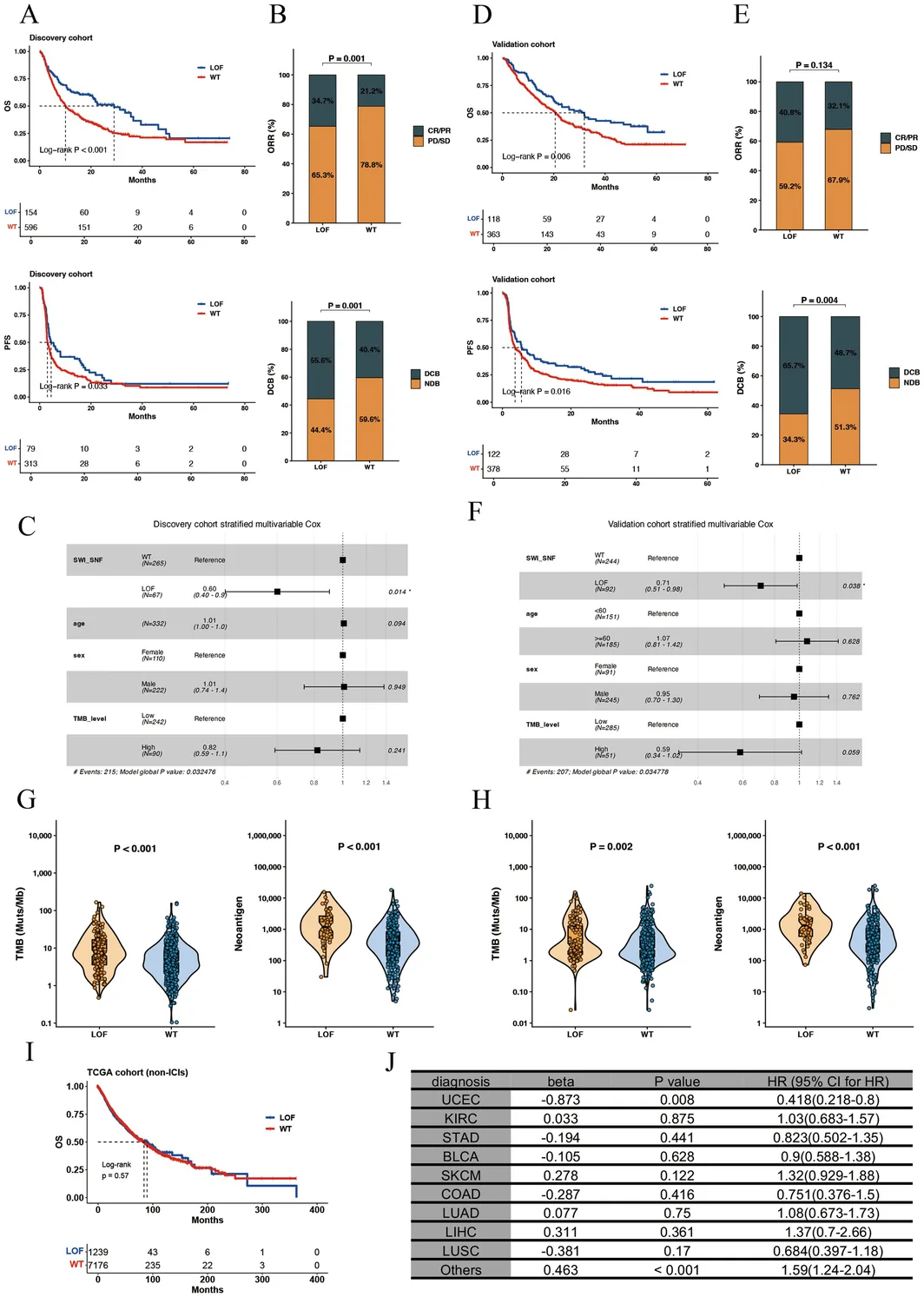
Clinical outcomes and multivariate analyses based on SWI/SNF‐LOF status in patients receiving immune checkpoint blockades (ICBs) in both the discovery and validation cohorts. A comparison of (A) overall survival (OS), progression‐free survival (PFS), (B) objective response rate (ORR), and durable clinical benefit (DCB) between SWI/SNF‐LOF and WT groups in the discovery cohort. (C) Multivariate Cox regression analyses of OS based on SWI/SNF‐LOF status in the discovery cohort. The same clinical outcomes analysed in the validation cohort, including (D) OS, PFS, (E) ORR, and DCB. (F) Multivariate Cox regression analyses of OS based on SWI/SNF‐LOF status in the validation cohort. (G, H) A comparison of tumour mutational burden (TMB) and neoantigen burden between SWI/SNF‐LOF and WT groups in both discovery and validation cohorts. (I) A comparison of overall survival (OS) between SWI/SNF‐LOF and WT groups in the TCGA cohort, and (J) a forest plot depicting subgroup analyses of different cancers in the validation cohort.

To evaluate whether LOF mutations in the SWI/SNF complex were associated with clinical benefit from ICB, we integrated clinical outcomes and whole‐exome sequencing data from four ICB‐treated cohorts, comprising 750 patients across five cancer types, as the discovery cohort (Figure S7A and Table S1). In this cohort, patients with SWI/SNF LOF mutations had significantly longer overall survival (OS) than WT patients (median OS, 29.0 vs. 10.1 months; HR, 0.60; 95% CI, 0.47–0.76; log‐rank *p* < 0.001; Figure 4A). Because Schoenfeld residual tests of the initial multivariable Cox model suggested a potential proportional hazards violation mainly related to the cancer type covariate (Table S3), subsequent multivariable analyses were performed using a Cox proportional hazards model stratified by cancer type. After adjustment for age, sex, and TMB level, SWI/SNF LOF remained independently associated with improved OS (HR, 0.60; 95% CI, 0.40–0.90; *p* = 0.014; Figure 4C). Schoenfeld residual tests showed no evidence of proportional hazards violation for the SWI/SNF term in the final stratified Cox model (Table S4). In addition, the SWI/SNF LOF group exhibited significantly longer progression‐free survival (PFS) than the WT group (median PFS, 4.5 vs. 3.0 months; HR, 0.73; 95% CI, 0.55–0.97; log‐rank *p* = 0.033; Figure 4A). Patients harbouring SWI/SNF LOF mutations also had higher objective response rate (ORR) and durable clinical benefit (DCB) rates (ORR, 34.7% vs. 21.2%; *p* = 0.001; DCB, 55.6% vs. 40.4%; *p* = 0.001; Figure 4B).

We next evaluated these findings in an independent validation cohort comprising 562 ICB‐treated patients across four cancer groups (Figure S7B and Table S2). Consistent with the discovery cohort, SWI/SNF LOF mutations were associated with significantly prolonged OS (median OS, 31.9 vs. 20.6 months; HR, 0.67; 95% CI, 0.50–0.89; log‐rank *p* = 0.006) and PFS (median PFS, 5.6 vs. 3.7 months; HR, 0.74; 95% CI, 0.58–0.95; log‐rank *p* = 0.016; Figure 4D). Using the same cancer type‐stratified multivariable Cox model, SWI/SNF LOF remained significantly associated with improved OS after adjustment for age group, sex, and TMB level (HR, 0.71; 95% CI, 0.51–0.98; *p* = 0.038; Figure 4F). Schoenfeld residual tests similarly showed no evidence of proportional hazards violation for the SWI/SNF term in the validation cohort model (Table S5). Patients with SWI/SNF LOF mutations also had a significantly higher DCB rate than WT patients (65.7% vs. 48.7%; *p* = 0.004), whereas the difference in ORR did not reach statistical significance (40.8% vs. 32.1%; *p* = 0.134; Figure 4E).

We next examined the consistency of the association between SWI/SNF LOF mutations and ICB benefit across individual cancer‐type cohorts. To reduce instability from sparse subgroup data, analyses were restricted to cohorts with ≥ 50 analysable patients and ≥ 10 patients with SWI/SNF LOF mutations. Across eligible cancer types, all available outcome measures that could support ICB benefit, including survival and response‐based endpoints, were evaluated. Overall, SWI/SNF LOF was consistently associated with favourable outcomes across cancer‐type cohorts, although the specific endpoints supporting this association varied by cancer type (Figure S8 and Table S6). Significant OS benefits were observed in KIRC, melanoma, GI, and BLCA, whereas significant PFS benefits were observed in KIRC, melanoma, GI, and NSCLC. Among cancer types with available ORR data, SWI/SNF LOF was associated with significantly higher ORR in melanoma and NSCLC and showed a favourable trend in KIRC. Among cancer types with available DCB data, SWI/SNF LOF showed numerically higher DCB rates in KIRC, melanoma, GI, and NSCLC, with statistical significance observed only in KIRC (*p* = 0.039). Together, these survival and response‐based findings indicate that the value of SWI/SNF LOF as a marker of ICB benefit is broadly consistent across cancer types, despite heterogeneity in the endpoints available or reaching statistical significance in each cohort.

To explore potential immunogenic features associated with SWI/SNF LOF, we compared TMB and neoantigen burden between SWI/SNF LOF and WT tumours. In both the discovery and validation cohorts, SWI/SNF LOF tumours had significantly higher TMB and neoantigen burden than WT tumours (discovery cohort, both *p* < 0.001; validation cohort, TMB *p* = 0.002 and neoantigen *p* < 0.001; Figure 4G,H). In addition, SWI/SNF LOF status was not significantly associated with OS in the TCGA non‐ICB cohort (Figure 4I,J). These results suggest that the association of SWI/SNF LOF with survival may be more specific to the ICB‐treated setting rather than reflecting a general prognostic effect.

Finally, we examined whether the predictive signal was specific to SWI/SNF LOF mutations rather than any SWI/SNF alteration. Compared with patients without SWI/SNF mutations, patients with SWI/SNF LOF mutations showed stronger associations with improved OS in both the discovery and validation cohorts (Figure S9A,C). By contrast, patients with non‐LOF SWI/SNF mutations showed weaker associations, and no significant PFS advantage was observed for non‐LOF SWI/SNF mutations compared with WT tumours (Figure S9B,D). These findings suggest that SWI/SNF LOF mutations may capture the ICB‐benefit‐associated signal more specifically than non‐LOF SWI/SNF alterations.

## 4 Discussion

In previous studies, the focus was on single genes or pre‐defined SWI/SNF complex genes [14, 17, 25, 26], whereas in this study, we analysed LOF mutations in all 29 SWI/SNF complex genes [12, 27], using WES data and the TCGA cohort.

The SWI/SNF chromatin remodelling complex is known to play a critical role in maintaining genome stability, and previous research has linked SWI/SNF complex mutations with higher TMB and MSI [16, 25]. Our study investigates the relationship between SWI/SNF LOF mutations and genomic immunogenicity across multiple types of cancer through a pan‐cancer analysis. We found that these mutations are associated with an increased TMB and neoantigen load, which may make tumours with these mutations more susceptible to immunotherapy. Additionally, we observed that SWI/SNF LOF mutations are associated with alterations in the expression of MHC genes, which play a critical role in antigen presentation and recognition by the immune system. In several types of cancer, particularly UCEC, STAD, and COAD, SWI/SNF LOF groups are commonly associated with a decreased CNV burden. It has been reported that ARID1A inactivation leads to telomere cohesion defects that selectively eliminate global chromosome aberrations during mitosis [41]. Therefore, this may be one of the reasons for the lower CNV burden in the SWI/SNF LOF group. The SWI/SNF LOF group may avoid genomic instability and reduce the risk of immune attack, while the WT group may be more vulnerable to genomic instability and immune escape. In summary, our study provides important insights into the role of SWI/SNF LOF mutations in tumour immunogenicity and chromosomal instability, with potential implications for the development of new therapeutic strategies.

Altered expression or mutations in SWI/SNF genes have been linked to increased infiltration of CD8^+^ T cells, enhanced sensitivity to T‐cell‐mediated cytotoxicity, and improved response to ICB therapy in certain cancer types [14, 37]. For instance, loss of PBAF function has been shown to increase the sensitivity of tumour cells to interferon‐γ (IFN‐γ) by upregulating *STAT1* and *IRF1* expression and increasing the secretion of chemokines such as *CXCL10* and *CCL5* [42]. *ARID1A* deficient tumour tissues showed a significant increase in immune cell infiltration, including CD8^+^ T cells and other immune cells [43]. Depletion of canonical BAF complex members, including *ARID1A*, resulted in T cells maintaining their effector program and reducing the expression of exhaustion‐related genes when infiltrating tumours. *ARID1A* depletion also limited the acquisition of exhaustion‐associated chromatin accessibility, ultimately improving the antitumor immune response [44]. Additionally, PD‐L1 expression was found to be higher in tumour tissues lacking *ARID1A* than in tumours expressing normal *ARID1A* levels [45]. Our study highlights that the pan‐cancer analysis aimed to uncover the mechanisms by which SWI/SNF LOF mutations modulate anti‐tumour immunity. Anti‐PD‐(L)1 and anti‐CTLA‐4 therapies are both dependent on cytotoxic T cells for boosting anti‐tumour immune response, which results from a series of crucial steps including T cell homing, priming, and exertion and maintenance of tumour killing activity. We found that the SWI/SNF LOF group had inflammatory patterns of immune activity in some cancer types, such as high levels of TIL region fraction, lymphocyte infiltration signature score or leukocyte fraction. Immune cell infiltration analysis conducted using CIBERSORT software and the expression analysis of T cytotoxic genes supported these findings. Our study explored the link between SWI/SNF LOF mutations and the mechanisms involved in the cancer immune response, specifically the upregulation of chemokines and receptors. These molecules are crucial in attracting and recruiting immune functional cells [25]. We found that the SWI/SNF LOF group showed an overexpression of immune checkpoints (PD‐L1, PD‐1, CTLA‐4, and LAG3) compared to the WT group in most cancer types. The activated antitumor immunity, high expression of immune checkpoints, and enhanced immunogenicity in the SWI/SNF LOF group suggest that these patients may benefit more from ICB treatment than the WT group. Combining ICB therapy with other targeted therapies that exploit SWI/SNF‐related pathways may therefore enhance treatment efficacy and address the problem of treatment resistance in SWI/SNF LOF tumours.

Recent preclinical and clinical studies have explored the potential association between SWI/SNF complex genomic alterations and the efficacy of ICB in multiple tumours [2, 3, 17]. In this study, analysis of the discovery cohort (*n* = 750) and validation cohort (*n* = 562) revealed that SWI/SNF LOF mutation status may serve as a valuable biomarker for predicting the efficacy of ICB therapy in multiple cancers, independent of other clinical and pathological factors. Although the carcinogenic effects of widely mutated SWI/SNF genes have been increasingly recognized in recent years [46], the specific functional impact of different alteration types remains poorly understood. Our results demonstrate that the SWI/SNF LOF mutations specifically predict favourable outcomes of ICB therapy, whereas non‐LOF alterations do not. This distinction may partly explain why some retrospective studies have failed to find a consistent association between SWI/SNF variants and ICB benefit [26], likely due to relatively small sample sizes or the use of predefined gene sets that may introduce bias and fail to fully represent the entire complex. As demonstrated in our study, precise definition of mutation type is a critical factor affecting the accuracy of such analyses.

This study has several limitations that should be taken into consideration. First, the availability of ICB‐treated cohorts with comprehensive clinical outcomes (OS, PFS, ORR, DCB) and complete mutation profiling of all SWI/SNF complex genes is inherently limited. As a result, our ICB cohort analyses were restricted to six cancer types. Although we performed extensive pan‐cancer immunogenomic analyses using the TCGA dataset to characterize the immunogenicity and the immune landscape associated with SWI/SNF LOF mutations across solid tumour types, the generalizability of our ICB‐related findings to cancer types not represented in these cohorts requires cautious interpretation and further validation. Second, the study did not include experimental validation to elucidate how SWI/SNF complex‐mediated chromatin remodelling alters immune cell function within the tumour microenvironment. Despite our efforts, no publicly available single‐cell sequencing datasets provided both high‐resolution transcriptomic profiles and matched comprehensive mutational data covering all SWI/SNF complex genes in LOF‐mutant tumours, precluding assessment of their impact on specific immune cell populations at single‐cell resolution in the current study. Future studies incorporating multi‐omics and functional approaches are warranted to further clarify the underlying mechanisms. Third, our analysis focused solely on SWI/SNF LOF mutations as a single biomarker for ICB response. It remains an open question whether combining SWI/SNF LOF status with other established or emerging biomarkers—such as PD‐L1 expression, CXCL13 levels, tumour mutational burden, or microsatellite instability—could further improve the accuracy of patient stratification for ICB therapy. Exploring such combinatorial biomarker strategies in future studies may help identify patient subpopulations that derive the greatest benefit from immunotherapy and refine the clinical utility of SWI/SNF LOF mutations as a predictive tool.

Despite these limitations, our findings have significant implications for the clinical evaluation of the efficacy of ICB therapy. Our results suggest that SWI/SNF LOF mutations could serve as a promising biomarker for predicting response to ICB therapy across multiple cancer types. Therefore, additional studies to further validate our findings and clarify the molecular mechanisms underlying these associations will be essential to advance our understanding of the role of SWI/SNF complex‐mediated chromatin remodelling in tumour immune evasion.

## 5 Conclusion

The SWI/SNF family of chromatin remodelers is among the most frequently mutated gene families in human cancers. In our study, we provide new insights into the mechanisms underlying this association by demonstrating that LOF mutations in SWI/SNF complexes are associated with elevated immunogenicity, enhanced infiltration of CD8^+^ T cells in the tumour microenvironment, and upregulation of gene signatures associated with immune activity. Importantly, our findings also suggest that SWI/SNF LOF mutations are associated with greater clinical benefit from ICB therapy compared to wild‐type counterparts across multiple cancer types, highlighting their potential as predictive biomarkers.

## Author Contributions

**Wei Zhang:** conceptualization, supervision. **Tengfei Zhu:** data curation, formal analysis. **Zixuan Tian:** data curation, formal analysis. **Ya‐nan Wang:** supervision, writing – review and editing. **Renji Liang:** writing – original draft. **Yi Lu:** data curation, formal analysis. **Zheng Yang:** writing – original draft.

## Funding

This work was supported by the National High Level Hospital Clinical Research Funding (Grant No. BJ‐2022‐144).

## Ethics Statement

This study does not involve ethical and moral constraints, and the data in this article are derived from public databases.

## Consent

The authors have nothing to report.

## Conflicts of Interest

Y.L. and T.Z. are employees of Burning Rock Biotech, Guangzhou, China. Other authors report there are no competing interests to declare.

## Supporting information


**Figure S1:** Distribution of loss‐of‐function mutations in SWI/SNF complexes in the TCGA pan‐cancer cohorts by (A) mutation class, (B) variant type, and (C) types of single‐nucleotide variation (SNV).

**Figure S2:** Lollipop plots showing site of loss‐of‐function mutations on each SWI/SNF subunit that harboured loss‐of‐function mutations in the TCGA cohorts.

**Figure S3:** Lollipop plots showing site of loss‐of‐function mutations on each SWI/SNF subunit that harboured loss‐of‐function mutations in the TCGA cohorts.

**Figure S4:** Lollipop plots showing site of loss‐of‐function mutations on each SWI/SNF subunit that harboured loss‐of‐function mutations in the TCGA cohorts.

**Figure S5:** Levels of CNV burden and homologous recombination defects (HRD) in SWI/SNF‐wild‐type (WT) vs. ‐loss‐of‐function (‐LOF) patients in the TCGA cohorts by tumour type. (A) Homologous Recombination Defects. (B) Number of Segments. (C) Number of LOH Segments.

**Figure S6:** A comparison of the expression patterns of immunoinhibitor between SWI/SNF‐LOF and WT groups in the TCGA cohorts by tumour type.

**Figure S7:** Flowchart of the process used for screening of the study population. (A) Flowchart of the process used for screening of patients included in the discovery cohort. (B) Flowchart of the process used for screening of patients included in the validation cohort.

**Figure S8:** A comparison of (A, C, E, G, I) overall survival (OS) and progression‐free survival (PFS), as well as (B, D, F, H) objective response rate (ORR) and durable clinical benefit (DCB) between SWI/SNF‐LOF and wild‐type (WT) groups of patients with different cancer types who were treated with immune checkpoint inhibitors (ICIs). The analyses of (A, B) kidney renal clear cell carcinoma (KIRC), (C, D) melanoma (Melanoma), (E, F) gastrointestinal (GI) cancer, (G, H) non‐small cell lung cancer (NSCLC), and (I) bladder cancer (BLCA) were presented.

**Figure S9:** Results of univariate Cox regression analyses of SWI/SNF mutation status on both overall survival (OS) and progression‐free survival (PFS). The analyses were conducted in the discovery cohort (A, OS; B, PFS) and the validation cohort (C, OS; D, PFS) of patients who received immune checkpoint inhibitor (ICB).

**Table S1:** Summarizes the clinical characteristics of 750 patients in the discovery cohort.

**Table S2:** Summarizes the clinical characteristics of 562 patients in the validation cohort.

**Table S3:** Schoenfeld residual tests for the initial multivariable Cox proportional hazards models before stratification by cancer type.

**Table S4:** Schoenfeld residual tests for the proportional hazards assumption of the stratified multivariable Cox proportional hazards model in the discovery cohort.

**Table S5:** Schoenfeld residual tests for the proportional hazards assumption of the stratified multivariable Cox proportional hazards model in the validation cohort.

**Table S6:** Cancer‐specific survival analyses according to SWI/SNF LOF mutation status.

## Data Availability

The datasets used and/or analysed during the current study are available from the corresponding author on reasonable request.
