# Supplementary material for: Loss‐of‐Function Mutations in SWI/SNF Complexes Associated With Active Tumour Immune Microenvironment Clinical Response to Immune Checkpoint Blockade
Source: J Cell Mol Med. 2026 Aug 25;30(16):e71290. doi: 10.1111/jcmm.71290 (PMC13507517; doi:10.1111/jcmm.71290)
Supplement: Supplementary file 1 [file JCMM-30-e71290-s001.docx]

Supplementary table 6. Cancer-specific survival analyses according to SWI/SNF LOF mutation status

| **Cancer type** | **Endpoint** | **N** | **Events** | **WT (n)** | **LOF (n)** | **HR (95% CI)** | **P value** |
| --- | --- | --- | --- | --- | --- | --- | --- |
| BLCA | OS | 374 | 246 | 296 | 78 | 0.65 (0.47–0.91) | 0.012 |
| GI | OS | 92 | 42 | 71 | 21 | 0.42 (0.18–0.95) | 0.038 |
| GI | PFS | 91 | 61 | 70 | 21 | 0.50 (0.26–0.96) | 0.036 |
| KIRC | OS | 261 | 181 | 167 | 94 | 0.66 (0.48–0.92) | 0.013 |
| KIRC | PFS | 261 | 221 | 167 | 94 | 0.75 (0.57–0.99) | 0.041 |
| Melanoma | OS | 442 | 261 | 372 | 70 | 0.58 (0.40–0.84) | 0.004 |
| Melanoma | PFS | 370 | 285 | 311 | 59 | 0.71 (0.51–1.00) | 0.049 |
| NSCLC | PFS | 131 | 93 | 112 | 19 | 0.44 (0.22–0.88) | 0.020 |

Cancer-specific analyses were performed using univariable Cox proportional hazards models. Cancer types with fewer than 50 patients were not included in the graphical subgroup presentation and should be interpreted with caution because of limited statistical power.
