# Supplementary material for: Loss‐of‐Function Mutations in SWI/SNF Complexes Associated With Active Tumour Immune Microenvironment Clinical Response to Immune Checkpoint Blockade
Source: J Cell Mol Med. 2026 Aug 25;30(16):e71290. doi: 10.1111/jcmm.71290 (PMC13507517; doi:10.1111/jcmm.71290)
Supplement: Supplementary file 2 [file JCMM-30-e71290-s003.docx]

Supplementary table 3. Schoenfeld residual tests for the initial multivariable Cox proportional hazards models before stratification by cancer type.

| **Cohort** | **Endpoint** | **Variable** | **χ²** | **df** | **P value** |
| --- | --- | --- | --- | --- | --- |
| Discovery | OS | SWI/SNF status | 1.222 | 1 | 0.269 |
| Discovery | OS | age | 2.092 | 1 | 0.148 |
| Discovery | OS | sex | 0.547 | 1 | 0.459 |
| Discovery | OS | cancer | 11.289 | 3 | 0.010 |
| Discovery | OS | TMB level | 1.224 | 1 | 0.268 |
| Discovery | OS | Global test | 18.163 | 7 | 0.011 |
| Validation | OS | SWI/SNF status | 0.390 | 1 | 0.532 |
| Validation | OS | age | 1.917 | 1 | 0.166 |
| Validation | OS | sex | 0.007 | 1 | 0.934 |
| Validation | OS | cancer | 3.664 | 2 | 0.160 |
| Validation | OS | TMB level | 0.406 | 1 | 0.524 |
| Validation | OS | Global test | 5.732 | 6 | 0.454 |

The proportional hazards assumption was evaluated using Schoenfeld residual tests for each covariate and for the global model in the discovery and validation cohorts.
