# Supplementary material for: Loss‐of‐Function Mutations in SWI/SNF Complexes Associated With Active Tumour Immune Microenvironment Clinical Response to Immune Checkpoint Blockade
Source: J Cell Mol Med. 2026 Aug 25;30(16):e71290. doi: 10.1111/jcmm.71290 (PMC13507517; doi:10.1111/jcmm.71290)
Supplement: Supplementary file 3 [file JCMM-30-e71290-s006.docx]

Supplemental table 2. Summarizes the clinical characteristics of 562 patients in the validation cohort.

|  | Overall (n = 562) |
| --- | --- |
| **sex** |  |
| Female | 195 (34.7%) |
| Male | 367 (65.3%) |
| **cancer** |  |
| GI | 92 (16.4%) |
| KIRC | 189 (33.6%) |
| Melanoma | 200 (35.6%) |
| NSCLC | 81 (14.4%) |
| **drug** |  |
| anti-PD-1/L1 | 469 (83.5%) |
| anti-CTLA-4 | 7 (1.2%) |
| combination | 86 (15.3%) |
| **cohort** |  |
| Checkmate025 | 189 (33.6%) |
| Hellmann_2018 | 75 (13.3%) |
| Hugo_2016 | 54 (9.6%) |
| Liu_2019 | 139 (24.7%) |
| PUCH_2021 | 92 (16.4%) |
| Rizvi_2015 | 6 (1.1%) |
| Synder_2014 | 7 (1.2%) |
