# Supplementary material for: Loss‐of‐Function Mutations in SWI/SNF Complexes Associated With Active Tumour Immune Microenvironment Clinical Response to Immune Checkpoint Blockade
Source: J Cell Mol Med. 2026 Aug 25;30(16):e71290. doi: 10.1111/jcmm.71290 (PMC13507517; doi:10.1111/jcmm.71290)
Supplement: Supplementary file 4 [file JCMM-30-e71290-s008.docx]

Supplemental table 1. Summarizes the clinical characteristics of 750 patients in the discovery cohort.

|  | Overall (n = 750) |
| --- | --- |
| **age (yr), Median [IQR]** |  |
| Median[IQR] | 62.00 [50.00, 71.00] |
| **sex** |  |
| Female | 213 (28.4%) |
| Male | 537 (71.6%) |
| **cancer** |  |
| NSCLC | 50 (6.7%) |
| BLCA | 374 (49.9%) |
| HNSCC | 12 (1.6%) |
| KIRC | 72 (9.6%) |
| Melanoma | 242 (32.3%) |
| **drug** |  |
| anti-PD-1/L1 | 504 (67.2%) |
| anti-CTLA-4 | 236 (31.5%) |
| combination | 10 (1.3%) |
| **cohort** |  |
| Allen_2015 | 110 (14.7%) |
| Checkmate009 | 35 (4.7%) |
| Checkmate010 | 37 (4.9%) |
| Miao_2018 | 221 (29.5%) |
| Imvigor210 | 347 (46.3%) |

IQR, interquartile range.
