# Supplementary material for: Loss‐of‐Function Mutations in SWI/SNF Complexes Associated With Active Tumour Immune Microenvironment Clinical Response to Immune Checkpoint Blockade
Source: J Cell Mol Med. 2026 Aug 25;30(16):e71290. doi: 10.1111/jcmm.71290 (PMC13507517; doi:10.1111/jcmm.71290)
Supplement: Supplementary file 5 [file JCMM-30-e71290-s009.docx]

Supplementary Table 4. Schoenfeld residual tests for the proportional hazards assumption of the stratified multivariable Cox proportional hazards model in the discovery cohort.

| **Cohort** | **Endpoint** | **Variable** | **χ²** | **df** | **P value** |
| --- | --- | --- | --- | --- | --- |
| Discovery | OS | SWI/SNF status | 0.114 | 1 | 0.736 |
| Discovery | OS | age | 3.575 | 1 | 0.059 |
| Discovery | OS | sex | 2.211 | 1 | 0.137 |
| Discovery | OS | TMB level | 5.077 | 1 | 0.024 |
| Discovery | OS | Global test | 8.76 | 4 | 0.067 |

The proportional hazards assumption was evaluated using Schoenfeld residual tests for each covariate and the global model in the stratified multivariable Cox proportional hazards model, with cancer type included as the stratification factor.
